# Phenol-Furfural Resin/Montmorillonite Based High-Pressure Green Composite from Renewable Feedstock (*Saccharum munja*) with Improved Thermo-Mechanical Properties

**DOI:** 10.3390/polym12071562

**Published:** 2020-07-14

**Authors:** Muhammad Zeeshan Asad, Azhar Mahmood, Syed Tasweer Hussain Shah

**Affiliations:** 1 School of Natural Sciences, National University of Sciences and Technology, H-12, Islamabad 44000, Pakistan; zeeshanasad062@gmail.com; 2 College of Electrical & Mechanical Engineering, National University of Sciences & Technology, Sector H-12, Islamabad 44000, Pakistan; tasweer@ceme.nust.edu.pk

**Keywords:** phenol-furfural resin, montmorillonite, saccharum munja, high-pressure composite

## Abstract

This research endeavour aimed to explore the potential of a native, nonedible and low market value plant feedstock, i.e., *Saccharum munja* for green synthesis of woodware materials and improve its features by incorporating an economical blending material. A significant amount of furfural, i.e., 58%, was extracted from *Saccharum munja* through the modified acid digestion method. Extracted furfural was reacted with phenol to prepare phenol-furfural resin, an alternative to phenol-formaldehyde resin but with no harmful effects for humans. The synthesized resin was also blended with montmorillonite clay after modification via Dimethyl Sulfoxide (DMSO) treatment for improved thermo-mechanical properties. These resins and composites were characterized by XRD, SEM, and FTIR spectroscopy. Resultant resins and composites were further employed as a binding agent to make high-pressure composite from leftover plant residue by hot-press method. The resultant product was subjected to TGA analysis and furnished high value of degradation temperature (*T*_deg_), i.e., 607 °C. Prepared high-pressure composite samples were mechanically tested through compression tests by Tinius Olsen Testing Machine and hardness tests by Rockwell Hardness Tester. Its tensile strength value was 58.3 MPa while hardness value was found to be 64 RHB which was greater than mild copper with hardness value 48.9 RHB. Thus, green high-pressure composite material was successfully developed by employing *Saccharum munja and* montmorillonite clay while no toxic resin was used, nor was any residue left over.

## 1. Introduction

The economy of the current world is affected by the shortage of various necessities of life, including food, shelter, clothing, domestic fuel, and other basic living articles because of the depletion of feedstock resources. Scientists have been working on various options to explore alternative ways to achieve better solutions to these basic needs. The desire to minimize the world’s dependence on fossil fuels has strengthened the interest in producing green chemicals, materials, and fuels from renewable feedstocks [1,2,3,4]. Plant wastes are good alternative feedstock for their ease of availability, economical cost, and renewable raw materials. *Saccharum munja* is a native nonedible plant with very low market value [5].

Phenol-formaldehyde (PF) resin, owing to its superb mechanical properties, chemical resistance, and thermal stability, has played an extensive role as engineered plastic in industry. PF resin is used on a large scale for the manufacturing of plywood where veneers or thin layers of wood are hot-pressed using PF as adhesive agents [6,7]. In the year 2004, the World Health Organization’s ‘International Agency for Research on Cancer’ marked formaldehyde as carcinogenic to humans. For this reason, PF resin should be replaced to avoid carcinogenic emission of formaldehyde [8]. This has driven the interest of various researchers for the substitution of PF resin with bio-based phenolic compounds, furfural, lignin and lignocellulosic biomass, etc. [9,10,11,12]. Furfural is a biomass derived-chemical that can be used to replace petrochemicals. Studies have been reported for conversion of pentoses [13,14,15] and hexoses [13] from biomass into furfural [16,17,18]. J. Yi et al. have investigated conversion of the hemicellulose in corn stover (stalk) by γ-Al_2_O_3_/SO_4_^2−^ solid acid catalyst under hydrothermal conditions at low temperature. This approach has selectively promoted the formation of furfural in the H_2_O-tetrahydrofuran solvent system [19]. Acidic 1-butyl-3-methylimidazolium hydrogen sulphate ionic liquid was employed by A. V. Carvalho et al. for the catalysis of the wheat straw biomass hemicellulose conversion into furfural and xylose. Temperature has demonstrated a greater effect on the production of xylose and furfural, rather than the time of pre-treatment, set at a fixed 1/10 (w/w) biomass/IL ratio and 1.24% (w/w) water content in the pre-treatment process [20]. Similar results were reported by P. Brazdausks and co-workers, who studied effect of the acid hydrolysis temperature and biomass pretreatment process time on the conversion of birch wood hemicelluloses into furfural at constant amount of catalyst loading, i.e., 3.0% [21,22]. Furfural has found its application in the manufacturing of green adhesive novolac-type PF resins owing to presence of aldehyde functional group and its extractability from renewable feedstock. Furfural is incorporated within the phenolic resin as furanylmethine and furanylmethylol groups [23,24]. A. Pizzi et al. have investigated the structure of traditional, linear phenol–resorcinol–formaldehyde (PRF) resins, urea-branched PRF resins, and phenol–resorcinol–furfural (PRFuran) resins. They found that very different percentages of resorcinol were needed for the equal performance of these resins as adhesives. PRF resin performance was improved by maximizing either the proportion of resorcinol-containing oligomers or methylol groups containing oligomers. However, in PRFuran resin, the determinant parameter is the higher molecular weight of furfural as compared to formaldehyde [25]. In another reported work, phenol-resorcinol-furfural, resorcinol-furfural, and resorcinol-phenol-furfural cold-setting resins were prepared to substitute formaldehyde-based cold-setting resins. The phenol-resorcinol-furfural adhesive resin has an advantage of lower volumetric shrinkage on curing [26]. F.B. Oliveira et al. have prepared resol type resins with furfural obtained by acid hydrolysis of abundant renewable resources from agricultural and forestry waste residues. Composites were prepared with furfural–phenol resins and sisal fibres without formaldehyde which showed excellent adhesion between resin and fibres [27]. Rapid curing of resin is desirable in many industrial applications. Increasing the ratio of furfural to phenol increases the speed of curing which may be attributed to the function of increasing molecular weight [28]. Fire and degradation behaviour can be improved by blending phenol-furfural resin with some clay materials [29]. Montmorillonite (MMT) clay is a hydrated sodium calcium aluminium magnesium silicate hydroxide (NaCa)_0.33_(AlMg)_2_(Si_4_O_10_)(OH)_2_·nH_2_O [30], having fundamental tetrahedral and octahedral sheets. MMT has a better heat-insulating property and thermal resistance when it is used as a stabilizer in a substance [31]. Curing behaviour and melt intercalation of phenolic resins can be improved by the introduction of layered silicates, such as pristine montmorillonite [32,33]. The improvement in the properties of materials can be achieved only when layers of clay are fully dispersed in the polymer matrix. Separated layers of clay offer smooth entrance of polymers into the galleries of clay. Surface of clay can be modified with different modifiers which promote the entrance of polymer into clay galleries [34]. In situ polymerization of phenolic resin/montmorillonite was performed by J. Pappas et al. It was found that auxiliary mixing of clay in phenol has promoted intercalation of oligomer and polymer between montmorillonite clay layers. Clay was predominantly exfoliated at 2.7% w/w and above this substantial amount of the clay aggregation was observed. Resultant composite was mechanically superior and exhibited thermal stability up to 200 °C [35]. L.B. Manfredi et al. have prepared composite of resol resin with the addition of modified and non-modified MMT clay via prepolymer intercalation method. The composites filled with the modified montmorillonites has shown a lower glass transition temperature value as well as a higher degradation peak at ~400 °C, which is characteristic of the degradation of methylene bridges, indicating a decrease in the crosslinking density of the resol network [36]. MMT clay has been applied as an inorganic synergist to prepare the water-based intumescent flame retardant (IFR) ornamental coating for plywood. Analysis of heated products has revealed that residual nitrogenous polyaromatic structure and residual mass in the IFR coating were the results of the effect of MMT on the antioxidation properties of the char layer [37].

In current work, phenol-furfural resin was synthesized by using furfural that was extracted from *Saccharum munja* plant. Resin was blended with organically modified montmorillonite clay to incorporate thermal stability. Resultant resin clay composite was mixed with plant leftover material and pressed at high temperature to manufacture high-pressure composite.

## 2. Materials and Methodology

### 2.1. Materials

*Saccharum munja* plant was collected from local fields of district DI Khan, Pakistan. The whole plant with leaves was shade dried for 10 days and ground to make powder. Subsequently crushed plant material was dried in an oven at 50 °C for a half-hour to completely remove moist contents. AlCl_3_·6H_2_O (99%, Daejung, Korea) NaCl (99%, Daejung, Korea), Chloroform (99%, Daejung, Korea), DMSO (99%, Daejung, Korea), HCl (37%, Daejung, Korea), Phenol (99%, Daejung, Korea) and KOH (99%, Daejung, Korea). Montmorillonite clay (99% pure) was purchased from Xi’an Sky Biological Technology China. All solutions were prepared in deionized water.

### 2.2. Extraction of Furfural from Saccharum Munja

A catalyst mixture consisting of an equal amount of AlCl_3_·6H_2_O and NaCl was prepared. An extraction medium was developed by preparing 2 w/v % catalyst material in 12% HCl solution. Plant powder was charged into this extraction medium in 1:10 proportion, mixed and stirred to ensure proper mixing of powder and extraction medium. Subsequently, this extraction system was poured into 1000 mL round bottom flask equipped with a condenser and heated at 100 °C for 2.5 h by an electrothermal heating mantle with constant stirring. These digested plant material mixtures were subjected to distillation and distillate was collected into the flask. The distillate was poured into separating funnel and chloroform was used as a separating solvent to extract the furfural from the distillate. Pure furfural was collected, weighed, and stored in an air-tight vial to prevent oxidation and evaporation. The higher yield (58%) of furfural from *Saccharum munja* was attributed to a higher percentage of pentosans in plant materials. Extracted furfural was pure as checked by TLC and used for resin synthesis without further purification.

### 2.3. Synthesis of Phenol-Furfural Resin

The phenol-furfural resin was prepared according to the method reported by J. Liu et al. [38]. Phenol and furfural were reacted in a 1:0.9 molar ratio. Phenol (12.5 g) was placed in a three-necked 100 mL round bottom flask and melted at 45 °C. KOH (0.25 g) was added as a catalyst into molten phenol and 11.5 g furfural was added dropwise up to 30 min with constant stirring. The reaction temperature was raised to 135 °C for 2 h that resulted in a dark fluid product. This was placed in a vacuum oven at a temperature of 135 °C under vacuum to remove unreacted phenol and to cease further polymerization. Subsequently, the solid dark colour phenol-furfural resin was obtained.

### 2.4. Exfoliation of Montmorillonite Clay

A quantity of 15 g of MMT clay was suspended into 300 mL DMSO solvent. This was heated at 80 °C with constant stirring for 5 days and sonicated for 30 min. Subsequently, washed with methanol several times and dried in the heating oven at 100 °C for 24 h. This organo modified MMT clay was ground to a fine powder.

### 2.5. Synthesis of MMT/Phenol-Furfural Composite

A quantity of 0.25 g organo modified MMT clay was added to 12.5 g molten phenol and stirred for an hour prior to the dropwise addition of 11.5 g furfural. After the complete reaction, the MMT-phenol-furfural composite was obtained. An attempt was made to add the maximum bearable amount of clay into phenol-furfural resin to increase the thermal stability and mechanical properties of ensuing composites. Only 2% clay with respect to molten phenol was used because at a lower percentage of clay, the reinforcement of clay and resin was also lower which may reduce thermal and mechanical properties of the resin. Similar results were observed by Mohan and Mettilda [39]. Contrary to this, when clay content was increased beyond 2%, mechanical stirrer could not completely mix the molten phenol and clay viscous suspension which resulted in nonhomogeneous composite and weak interfacial interaction between polymer and clay.

### 2.6. Manufacturing of High Pressure Composite

**Figure 1:**
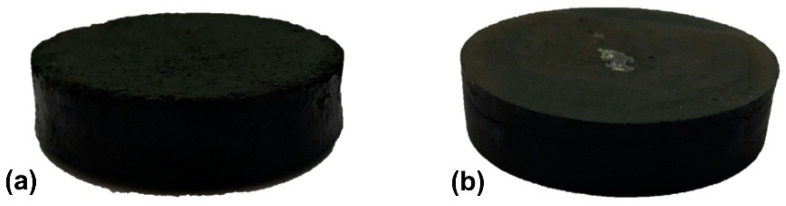
High-pressure composite prepared from plant residue and: (**a**) Phenol-furfural resin; (**b**) Phenol-furfural resin/organo modified montmorillonite (MMT) clay composite.

High-pressure composite was manufactured by mixing phenol-furfural resin with plant residue that left after extraction of furfural in a 1:3 ratio. The mixture was roasted at 135 °C for a half-hour to evenly mix residue with resin. Afterward, it was pressed in Metkon Ecopress 50 Mounting press at 135 °C for 1 h under a pressure of 40–50 atm. A round-shaped and 22 mm thick high-pressure composite was obtained. A similar procedure was repeated for the manufacturing of high-pressure composite from phenol-furfural/MMT composites (Figure 1).

## 3. Results and Discussion

### 3.1. FTIR Results

**Figure 2:**
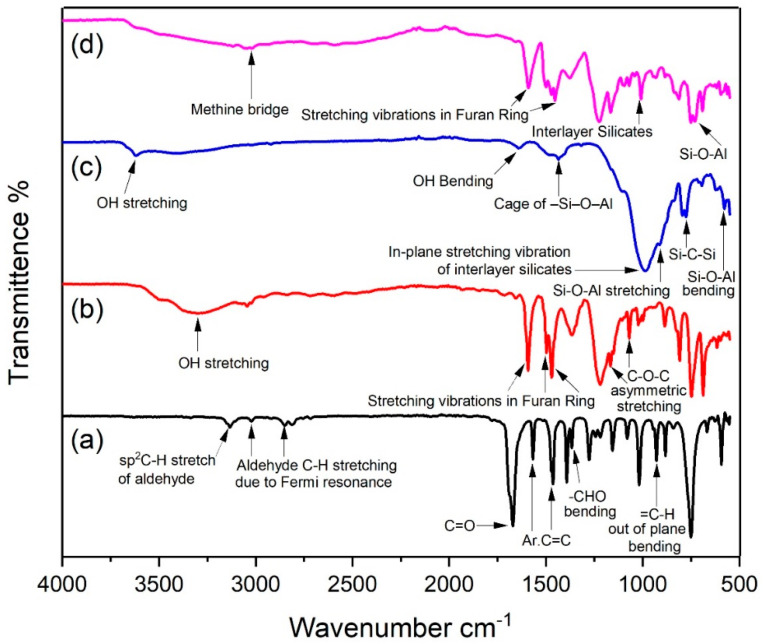
FTIR spectra of: (**a**) Furfural extracted from *Saccharum munja;* (**b**) Phenol-furfural resin; (**c**) Organo modified MMT clay; (**d**) Phenol-furfural resin/organo modified MMT clay composite.

The structure of furfural was corroborated by the existence of band at 3133 cm^−1^ in the FTIR spectrum which is a characteristic sp^2^ C–H bond stretch of aldehyde (Figure 2a). The presence of two bands at 3021 cm^−1^ and 2852 cm^−1^ have further confirmed the aldehyde group of furfural [40]. These two bands showed intense C–H stretching of aldehyde resulted from Fermi resonance related to the first overtone of the bending vibration at 1364 cm^−1^. A strong band related to a conjugated carbonyl group (C=O) was exhibited at 1674 cm^−1^. Two strong bands observed at 1569 cm^−1^ and 1466 cm^−1^ are revealing C=C bond showing aromatic ring, while =C–H out of plane bending appeared at 930 cm^−1^. C–O stretching vibrations were observed as a strong band at 1127 cm^−1^ [41]. For resin the absorption at 3308 cm^−1^ was assigned to –OH vibrations (Figure 2b). Bands at 1591 cm^−1^, 1498 cm^−1^ and 1470 cm^−1^ have denoted aromatic furan rings that show stretching vibrations in furan ring. The absorption bands at 1170 cm^−1^ and 1067 cm^−1^ were related to C–O–C asymmetrical stretching vibrations. The FTIR spectrum of organo-modified montmorillonite (Figure 2c) has depicted a broad band at 3624 cm^−1^ corresponds to –OH stretching vibration of inter-layer water while the band at 1639 cm^−1^ was related to –OH bending stretch of water adsorbed by clay. Similar results were mentioned elsewhere [42]. The spectrum band at 1431 cm^−1^ showed a cage of –Si–O–Al. In-plane stretching vibration of interlayer silicates was assigned to absorption at 991 cm^−1^. The Si–O–Al stretching and bending vibrations in montmorillonite were found at 914 cm^−1^ and 576 cm^−1^ respectively [43]. The symmetric stretching band of Si–C–Si was observed at 774 cm^−1^.

The chemical structure of the resin clay composite was studied by FTIR (Figure 2d) and the influence of clay addition to the resin structure was also analysed. It was anticipated that the presence of clay particles will promote a higher crosslinked chemical structure [44], which later leads to increment in the hardness value and better thermal resistance than bare resin. The resultant crosslinked structure also entered the matrix chains into the clay galleries and consequently, the intercalation or exfoliation of the montmorillonite. It was observed that, during cross-linking reaction, methine bridges were formed, which demonstrated absorption at 3018 cm^−1^. However, this was not so high, therefore this was not the main crosslink bridging factor. Since composite has shown higher crosslinking density, it exhibited low OH band signals. These findings accorded with those of other researchers [45]. This could be explained by the presence of Na^+^ ions, which can form chelates, thus promoting the addition reaction between furfural and phenol. This lowers the activation energy which resulted in higher polymerization.

### 3.2. Particle Size Analysis

**Figure 3:**
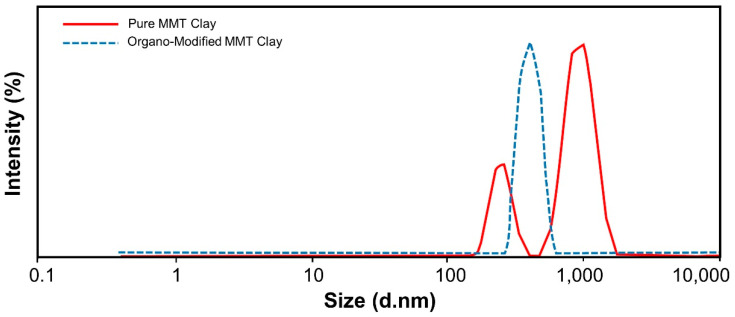
Particle size analysis of pure montmorillonite clay and organo modified MMT clay.

Particle size analysis was performed with Malvern Zeta-Sizer (Malvern Instruments Ltd., Worcestershire, UK). The dispersion solution was prepared by dispersing 1 mg pure clay and 1 mg modified organo clay into 1 mL de-ionized water separately. A volume of 1 µL of these dispersion solutions were further diluted into 1 mL of de-ionized water having final pH 7.0. Zeta size was measured at 25 °C with a count rate of 285.4 kcps. Figure 3 has shown the zeta-size results of untreated clay. Two peaks at 932.2 nm and 246.0 nm were recorded with 74.8% and 25.2% intensities respectively that showed that clay particles existed in aggregates form. Their Z-average value was 856.8 nm. Particle size analysis of organo modified clay has furnished the single peak at 400.2 nm with 100% intensity (Table 1). The Z-average value for organo treated clay was reduced to 669.9 nm, showing a reduction in particle size after treatment (Figure 3).

**Figure 4:**
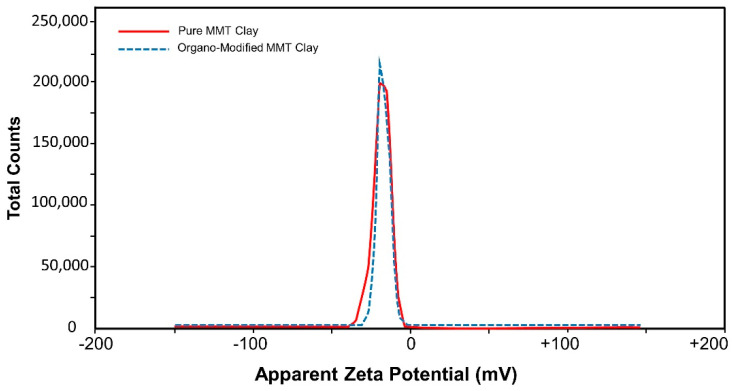
Zeta potential of pure montmorillonite clay and organo modified MMT clay.

Zeta potential of both pure and treated clay samples was measured at dispersion pH 7.0. The zeta potential of pure clay was found to be −19.2 mV with 100% area while the zeta-potential of treated clay was reduced to −17.6 mV with 100% area (Table 2, Figure 4). Zeta potential reduction to less negative values may be attributed to the fact that organic cations were adsorbed on the clay [46].

### 3.3. XRD Results

**Figure 5:**
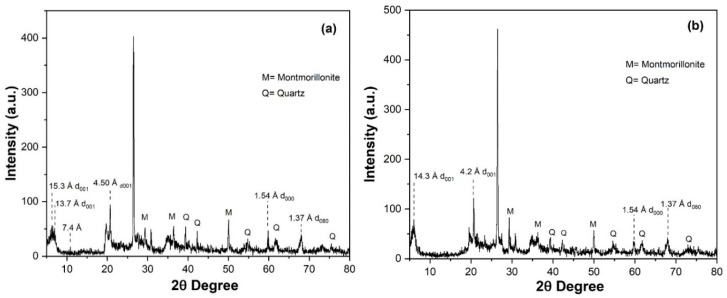
XRD pattern of: (**a**) Untreated montmorillonite clay; (**b**) Organo modified MMT clay.

Figure 5a has depicted the XRD pattern of untreated MMT clay. Interlayer Ca^2+^ and Mg^2+^ were characterized by their characteristic peaks with d-spacing d_001_ 15.3 Å while Ca^2+^, Mg^2+^ and Na^+^ were characterized by d_001_ 13.7 Å. The presence of Na^+^ specie has confirmed the montmorillonite clay. A peak at 7.4 Å has shown the interlayer spacing in the clay. The peak in 001 plane at angle 19.033 was associated with the basal spacing of 4.50. Dioctahederal structure of montmorillonite was corroborated by d-spacing 1.37 Å at plane 080. In Figure 5, some peaks were labelled as M and Q, which are associated with the montmorillonite and quartz phases, respectively.

Figure 5b has shown the XRD spectrum of organo-modified clay. Introduction of organic moieties in the clay shifted the peak at plane 15.3 Å to 14.3 Å. The plane d_001_ with d-spacing 4.50 Å was also shifted to 4.2 Å. However, the peak at the plane (080) persisted, which showed that the dioctahederal structure of clay was not disturbed by the introduction of organic phases.

**Figure 6:**
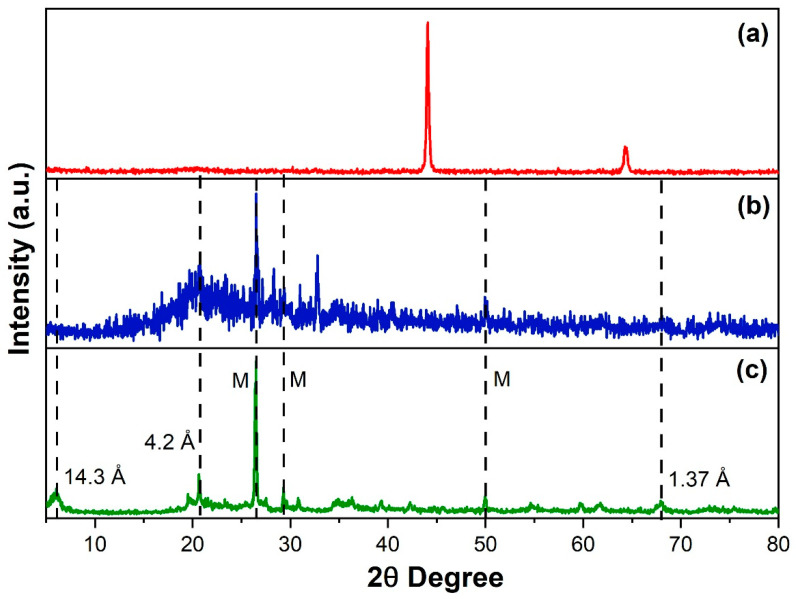
XRD results of: (**a**) Phenol-furfural resin; (**b**) Phenol-furfural resin/organo modified MMT clay composite; (**c**) Organo modified MMT clay.

XRD results of pure resin and clay blended resins were compared to study the effect of clay blending into a composite (Figure 6). The peak at 4.2 Å has shown that –OH groups in clay were bonded to polymeric resin which confirmed that resin has entered the galleries of the clay. These shifting of composite angles into higher values may be attributed to the fact that clay modifiers were displaced from the galleries. During polymerization, polymeric resin monomers have come close together with the galleries of the clay thus occupy the inside and outside spaces of clay. When the resin was cured, monomers turned into oligomers and finally into polymers, resulting in the dispersion of clay in the resin matrix. These results were obtained only when clay was mixed in situ.

### 3.4. SEM Results

**Figure 7:**
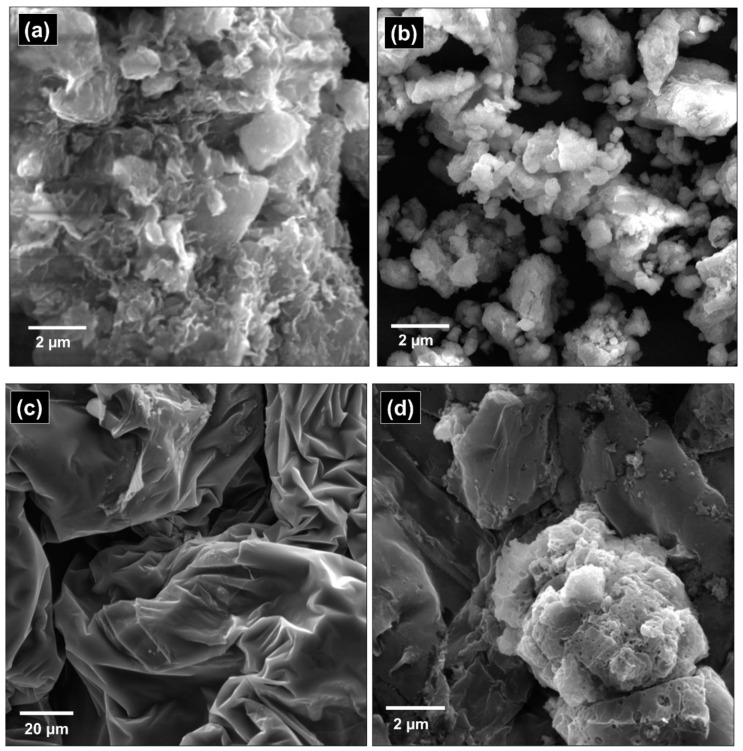
SEM images of: (**a**) Pure montmorillonite clay; (**b**) Organo-modified montmorillonite clay; (**c**) Phenol furfural resin; (**d**) Phenol-furfural resin/organo modified MMT clay composite.

SEM analysis was performed by using SEM VEGA3 LMU at an accelerating voltage of 20.00 Kv. SEM micrographs of untreated MMT clay have depicted larger particles that were aggregated in the form of flakes (Figure 7a). This has a layered structure with different size flakes and the range of particle size was 700–900 nm. The surface of untreated MMT clay was not smooth and have non-homogenous dispersion of particles. SEM images have also exhibited pores that were randomly distributed over a wide range of different sizes. These findings coincided with the results of other researchers in the literature [47,48]. Figure 7b has illustrated SEM results of MMT clay treated with DMSO. This has shown fine particles of MMT clay because of reduced particle size by exfoliation. The layering of MMT clay persisted while the even distribution of particles occurred. Particle size was reduced to 600 nm and larger aggregates disappeared or only very few were present. Larger flakes of MMT clay also disappeared after treatment with DMSO. This suggests a reduction of particle size. Similar results were reported in the literature for kaolinite clay treated with DMSO [49]. Since the layered structure of clay did not vanish, only cations were displaced by DMSO and no other structural changes occurred in clay. Figure 7c demonstrated the SEM micrographs of phenol-furfural resin. Branched micro-structures of resin have shown that polymerization between phenol and furfural has been propagated in a pattern similar to the 3D network. A high degree of branching also suggested that resin has no unreacted phenol residue and does not require any curing agent for efficient polymerization. This branching morphology has also been imparted thermosetting behaviour and crosslinking properties in the resulting resin.

Figure 7d has depicted the SEM results of phenol-furfural MMT composite. SEM micrographs have indicated the dispersion of clay on the surface of the resin. A boundary can be seen between clay particles (lighter region) and resin surface (dark region). The lighter region was an outcome of clay conductive behaviour after gold sputtering.

### 3.5. Thermogravimetric Analysis

**Figure 8:**
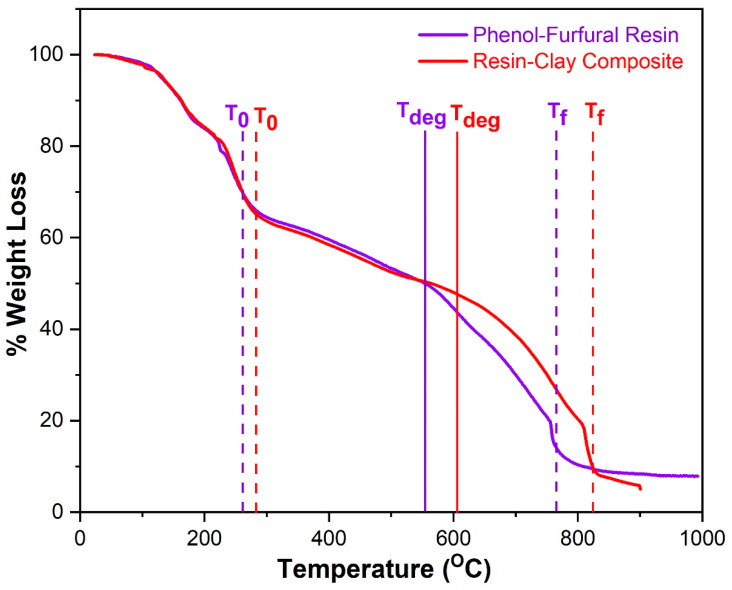
TGA thermogram of Phenol-furfural (*Munja*) resin and Phenol-furfural resin/organo modified MMT clay composite.

TGA analysis was executed by the DTA-TGA-50/50H SHIMADZU machine. The temperature profile range was set from 20 °C to 1000 °C under the nitrogen atmosphere. Both pure resins and composites were tested for their thermal stability. Pure *Saccharum munja* resin started decomposition at a temperature of 266 °C denoted by T_0_ while its decomposition was completed at temperature of 765 °C denoted by *T*_f_ (Figure 8). Decomposition peak temperature, where the decomposition is at the maximum, was 550 °C denoted by *T*_deg_.

Figure 8 has also depicted the thermogram of *Saccharum munja* resin plus MMT clay composite. Decomposition was started at 280 °C and completed at 828 °C while decomposition peak temperature was recorded at 607 °C (Table 3). Comparison of TGA results has shown that onset, endset and decomposition peak temperatures have been increased upon the incorporation of clay in resin which resulted in higher thermal stability. Similar results were obtained for a conventional phenolic resin [50]. The temperature up to 280 °C could be related to the removal of residual monomer, oligomer, and water evaporation. Temperature regions from 280 °C to 828 °C gave overall degradation, where breaking of chains and reactions related to crosslinking occurred. Hence, the previously formed structure partially decomposes, and carbonaceous residue is formed at temperatures beyond 828 °C. Thermogravimetric results have shown that introduction of clay into pure resin has reduced the rate of degradation, which could be related to the more crosslinked structure of clay and resin.

### 3.6. Mechanical Testing

#### 3.6.1. Compression Test

**Figure 9:**
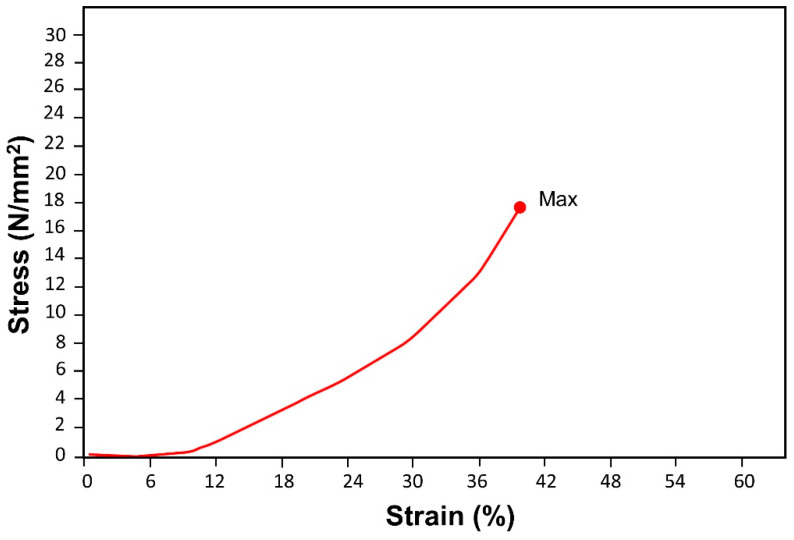
Compression test graph of phenol-furfural resin/organo modified MMT clay based high-pressure composite by UTM.

A compression test was performed by the Universal Testing Machine (UTM). Compression was forced at a speed of 2 mm/min for high-pressure composite sample that was made from leftover plant residue of *Saccharum munja* with phenol-furfural/MMT clay composite. UTM has applied its maximum force of compression, i.e., 20 KN but there was no breakage in the high-pressure composite. Measured values were maximum force 20,000 N, maximum stress 12.6450 N/mm^2^ and maximum strain 39.6358% at the entire test area of the sample (Table 4). All these values have shown that composite material was strongly bonded with plant residue filling material so that it was tough enough to bear 20,000 N force of compression (Figure 9).

**Figure 10:**
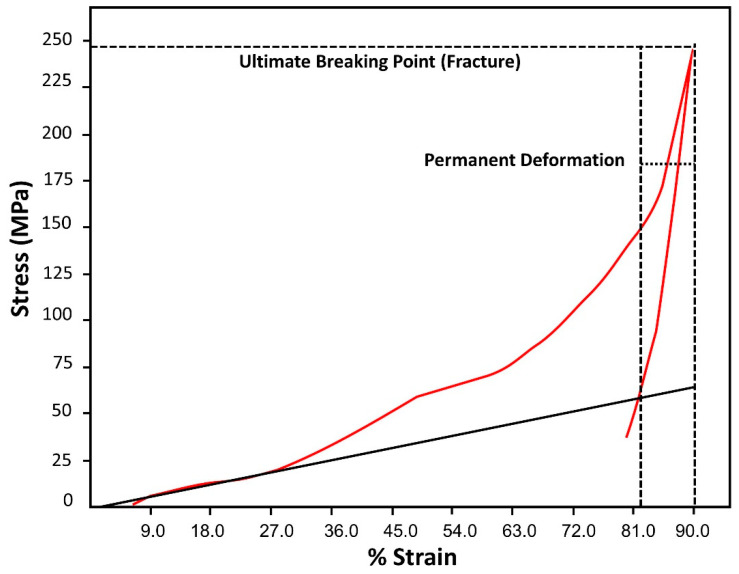
Compression test graph of phenol-furfural resin/organo modified MMT clay composite based high pressure composite by Tinius Olsen Press.

In order to study the breaking point of high pressure composite another compression machine, Tinius Olsen was employed with a maximum compression force of 300 KN. High-pressure composite sample was broken at the ultimate breaking point of 246 MPa. Its measured stress value was 58.3 MPa and tensile strain was 74% (Figure 10). These results indicated good mechanical strength of tested high-pressure composite samples as compared to commercial plywood with the ultimate tensile strength of 31 MPa (ASTM D3500) [51]. Greater compression behaviour is credited to the intercalated clay particles which promoted better matrix and mechanical caging. As a result, increased binding strength was exhibited between plant residue and clay modified resin. This phenomenon of clay cage architecture has helped to improve compression property [52].

#### 3.6.2. Rockwell Hardness Testing

The hardness of high-pressure composite was measured through Rockwell Hardness Tester, which furnished hardness values of material via extant of dent caused by shaft penetration driven by control loads. The penetration of dent was noted through the dial which showed scales of hardness. A small initial weight of 10 Kg was applied, followed by the main load. The hardness of high-pressure composite was founded 64 RHB at Rockwell Hardness B Scale. This scale is used to test softer metals including aluminium, brass, and soft steel. Comparisons of hardness values of high-pressure composite samples with other metals were tabulated in Table 5.

## 4. Conclusions

A native, nonedible, economical, and renewable material, i.e., *Saccharum munja* plant was successfully employed to extract furfural for the synthesis of green resin that served as filling material for high-pressure composite manufacturing so that no residual material was thrown into the environment. Synthesized furfural-based green resin is an alternative of formaldehyde-based carcinogenic resin. The resin was made thermally stable by incorporating commonly available MMT clay after modification via DMSO treatment. Green high-pressure composite was prepared from resin-clay composite and plant residue leftover material. TGA analysis, mechanical, and hardness tests have revealed that this green high-pressure composite possessed improved thermo-mechanical features as compared to commercial plywood materials. More crosslinking of polymer and clay suggested the diminished rate of degradation, where compression behaviour was improved by addition of clay for its better matrix and mechanical caging.

## Figures and Tables

**Table 1:** Particle size analysis of untreated and organo modified MMT clay.

Sample	Signal	Size (d.nm)	% Intensity	Std Deviation	Z-Average
**Untreated Clay**	Peak 1	932.2	74.8	226.3	856.8 d.nm
	Peak 2	246.0	25.2	45.53	
**Organo-Modified Clay**	Peak 1	400.2	100	66.13	669.9 d.nm

**Table 2:** Zeta potential of untreated and organo modified MMT clay.

Sample	Signal	Mean	Area	Std Deviation	Zeta Potential Avg.
**Untreated Clay**	Peak 1	−19.2 mV	100 %	5.74 mV	−19.2 mV
**Organo Modified Clay**	Peak 1	−17.6 mV	100 %	4.07 mV	−17.6 mV

**Table 3:** TGA results of phenol-furfural (*Munja)* resin and phenol-furfural (*Munja)* resin/organo modified MMT clay composite.

Samples	* T_0_	*T*_f_	*T*_deg_	Increment
**Pure *munja* resin**	266 °C	765 °C	550 °C	-
***Munja*** **resin clay composite**	280 °C	828 °C	607 °C	57 °C

* *T*_0_ = Onset Temperature, *T*_f_ = Endset Temperature, *T*_deg_ = Decomposition Peak Temperature.

**Table 4:** Compression test results of phenol-furfural/MMT clay based high-pressure composite by UTM.

Sample	Max_Force * (N)	Max_Stress (N/mm^2^)	Max_Disp. (mm)	Max_Strain (Areas %)
*Saccharum munja* plywood	20,017.0	17.6450	8.32352	39.6358

* Calculated for entire test area of the sample.

**Table 5:** Comparison of hardness values of phenol-furfural resin/organo modified MMT clay based high-pressure composite on RHB Scale.

Material	RHB Values
High-Pressure Sample	64.0
Copper	48.9
Brass	63.2
Aluminium 6061 T6	63.5
Cast Iron	95.9
Low Carbon Steel	91.3

## References

[1] Huber G.W., Iborra S., Corma A. (2006). Synthesis of transportation fuels from biomass: Chemistry, catalysts, and engineering. Chem. Rev..

[2] Huber G.W., Chheda J.N., Barrett C.J., Dumesic J.A. (2005). Production of liquid alkanes by aqueous-phase processing of biomass-derived carbohydrates. Science.

[3] Tondi G., Schnabel T. (2020). Bio-Based Polymers for Engineered Green Materials. Polymers.

[4] Zhang Y., Heo Y.-J., Park M., Park S.-J. (2019). Recent advances in organic thermoelectric materials: Principle mechanisms and emerging carbon-based green energy materials. Polymers.

[5] Vasudevan P., Gujral G., Madan M. (1984). Saccharum munja Roxb., an underexploited weed. Biomass.

[6] Wu Z., Xi X., Lei H., Liang J., Liao J., Du G. (2019). Study on Soy-Based Adhesives Enhanced by Phenol Formaldehyde Cross-Linker. Polymers.

[7] Li Z., Zhou W., Yang L., Chen P., Yan C., Cai C., Li H., Li L., Shi Y. (2019). Glass fiber-reinforced phenol formaldehyde resin-based electrical insulating composites fabricated by selective laser sintering. Polymers.

[8] Kurple K.R. (1989). Foundry Resins. U.S. Patent.

[9] Cheng S., Yuan Z., Leitch M., Anderson M., Xu C.C. (2013). Highly efficient de-polymerization of organosolv lignin using a catalytic hydrothermal process and production of phenolic resins/adhesives with the depolymerized lignin as a substitute for phenol at a high substitution ratio. Ind. Crop. Prod..

[10] Wang M., Leitch M., Xu C.C. (2009). Synthesis of phenol–formaldehyde resol resins using organosolv pine lignins. Eur. Polym. J..

[11] Wang M., Xu C.C., Leitch M. (2009). Liquefaction of cornstalk in hot-compressed phenol–water medium to phenolic feedstock for the synthesis of phenol–formaldehyde resin. Bioresour. Technol..

[12] Zhang S., Chen H., Pizzi A., Li Y., Gao Q., Li J. (2014). Characterization and application of urea-formaldehyde-furfural co-condensed resins as wood adhesives. BioResources.

[13] Lü X., Saka S. (2012). New insights on monosaccharides’ isomerization, dehydration and fragmentation in hot-compressed water. J. Supercrit. Fluids.

[14] Garrett E.R., Dvorchik B.H. (1969). Kinetics and mechanisms of the acid degradation of the aldopentoses to furfural. J. Pharm. Sci..

[15] Ahmad T., Kenne L., Olsson K., Theander O. (1995). The formation of 2-furaldehyde and formic acid from pentoses in slightly acidic deuterium oxide studied by 1H NMR spectroscopy. Carbohydr. Res..

[16] Zhang T., Kumar R., Wyman C.E. (2013). Enhanced yields of furfural and other products by simultaneous solvent extraction during thermochemical treatment of cellulosic biomass. RSC Adv..

[17] Schuster K.C., Rohrer C., Eichinger D., Schmidtbauer J., Aldred P., Firgo H. (2004). Environmentally friendly lyocell fibers. Natural Fibers, Plastics and Composites.

[18] Lehnen R., Saake B., Nimz H.H. (2001). Furfural and hydroxymethylfurfural as by-products of FORMACELL pulping. Holzforschung.

[19] Yi J., He T., Jiang Z., Li J., Hu C. (2013). AlCl3 catalyzed conversion of hemicellulose in corn stover. Chin. J. Catal..

[20] Carvalho A.V., da Lopes A.M., Bogel-Łukasik R. (2015). Relevance of the acidic 1-butyl-3-methylimidazolium hydrogen sulphate ionic liquid in the selective catalysis of the biomass hemicellulose fraction. RSC Adv..

[21] Brazdausks P., Vedernikovs N., Puke M., Kruma I. (2014). Effect of the Acid Hydrolysis Temperature on the Conversion of Birch Wood Hemicelluloses into Furfural. in Key Engineering Materials. Key Eng. Mater..

[22] Brazdausks P., Puke M., Vederņikovs N., Krūma I. (2013). Influence of biomass pretreatment process time on furfural extraction from birch wood. Environ. Clim. Technol..

[23] Kim M.G., Boyd G., Strickland R. (1994). Adhesive properties of furfural-modified phenol-formaldehyde resins as oriented strandboard binders. Holzforschung.

[24] Brown L.H. (1952). Resin forming reactions of furfural and phenol. Ind. Eng. Chem..

[25] Pizzi A., Pasch H., Simon C., Rode K. (2004). Structure of resorcinol, phenol, and furan resins by MALDI-TOF mass spectrometry and 13C NMR. J. Appl. Polym. Sci..

[26] Pizzi A., Orovan E., Cameron F. (1984). The development of weather-and boil-proof phenol-resorcinol-furfural cold-setting adhesives. Holz als Roh Werkst..

[27] Oliveira F.B., Gardrat C., Enjalbal C., Frollini E., Castellan A. (2008). Phenol–furfural resins to elaborate composites reinforced with sisal fibers—Molecular analysis of resin and properties of composites. J. Appl. Polym. Sci..

[28] Brown L., Watson D. (1959). Curing phenol-furfural resins. Ind. Eng. Chem..

[29] Rivero G., Villanueva S., Manfredi L.B. (2014). Furan resin as a replacement of phenolics: Influence of the clay addition on its thermal degradation and fire behaviour. Fire Mater..

[30] Uddin F. (2018). Montmorillonite: An introduction to properties and utilization. Current Topics in the Utilization of Clay in Industrial and Medical Applications.

[31] Uddin F. (2008). Clays, nanoclays, and montmorillonite minerals. Metall. Mater. Trans. A.

[32] Byun H.Y., Choi M.H., Chung I.J. (2001). Synthesis and characterization of resol type phenolic resin/layered silicate nanocomposites. Chem. Mater..

[33] Kato M., Tsukigase A., Usuki A., Shimo T., Yazawa H. (2006). Preparation and thermal properties of resole-type phenol resin–clay nanocomposites. J. Appl. Polym. Sci..

[34] Choi M.H., Chung I.J., Lee J.D. (2000). Morphology and curing behaviors of phenolic resin-layered silicate nanocomposites prepared by melt intercalation. Chem. Mater..

[35] Pappas J., Patel K., Nauman E. (2005). Structure and properties of phenolic resin/nanoclay composites synthesized by in situ polymerization. J. Appl. Polym. Sci..

[36] Manfredi L.B., Puglia D., Kenny J.M., Vázquez A. (2007). Structure-properties relationship in resol/montmorillonite nanocomposites. J. Appl. Polym. Sci..

[37] Hu X., Sun Z., Zhu X., Sun Z. (2020). Montmorillonite-synergized water-based intumescent flame retardant coating for plywood. Coatings.

[38] Liu J., Wang J., Fu Y., Chang J. (2016). Synthesis and characterization of phenol–furfural resins using lignin modified by a low transition temperature mixture. RSC Adv..

[39] Mohan Babu K., Mettilda M. (2014). Studies on mechanical, thermal, and morphological properties of glass fibre reinforced polyoxymethylene nanocomposite. J. Appl. Chem..

[40] Purbowatiningrum R.S., Hapsari M., Rafi’ah F.H., Haq M.S. (2017). Synthesis of Furfural from Water Hyacinth (Eichornia croassipes). IOP Conference Series: Materials Science and Engineering.

[41] Nsubuga H., Chanbasha B., Al-Muallem H.A.S., Kalanthoden A.N. (2016). Isolation, characterization and evaluation of photochemical potential of rice husk-based furfural via continuous flow reactor. J. Environ. Chem. Eng..

[42] Hu X., Zhu X., Sun Z. (2019). Efficient flame-retardant and smoke-suppression properties of MgAlCO3-LDHs on the intumescent fire retardant coating for steel structures. Prog. Org. Coat..

[43] Akyüz S., Akyüz T., Yakar A. (2001). FT-IR spectroscopic investigation of adsorption of 3-aminopyridine on sepiolite and montmorillonite from Anatolia. J. Mol. Struct..

[44] Hu X., Zhu X., Sun Z. (2018). Effect of CaAlCO3-LDHs on fire resistant properties of intumescent fireproof coatings for steel structure. Appl. Surf. Sci..

[45] Manfredi L.B., Puglia D., Tomasucci A., Kenny J.M., Vázquez A. (2008). Influence of clay modification on the properties of resol nanocomposites. Macromol. Mater. Eng..

[46] Zadaka D., Radian A., Mishael Y.G. (2010). Applying zeta potential measurements to characterize the adsorption on montmorillonite of organic cations as monomers, micelles, or polymers. J. Colloid Interface Sci..

[47] Grim R.E. (1968). Clay Mineralogy.

[48] Karthikeyan G., Pius A., Alagumuthu G. (2005). Fluoride Adsorption Studies of Montmorillonite Clay. Indian J. Chem. Technol..

[49] Zulfiqar S., Sarwar M.I., Rasheed N., Yavuz C.T. (2015). Influence of interlayer functionalization of kaolinite on property profile of copolymer nanocomposites. Appl. Clay Sci..

[50] Puglia D., Kenny J.M., Manfredi L.B., Vázquez A. (2001). Influence of the chemical composition on the thermal degradation and fire resistance of resol type phenolic resins. Mater. Eng. Modena.

[51] ASTM D3500 (2014). Standard Test Methods for Structural Panels in Tension.

[52] Kumar M.S.S., Raju N.M.S., Sampath P.S., Selvan M.C.P. (2018). Influence of nanoclay on mechanical and thermal properties of glass fiber reinforced polymer nanocomposites. Polym. Compos..

